# Patient-reported outcomes in tuberculosis: a qualitative exploration of psychosocial, economic, and treatment-related challenges

**DOI:** 10.36416/1806-3756/e20250159

**Published:** 2025-09-22

**Authors:** Pedro Viegas, Luís L Ferreira, Mariana Vieira, Pedro Barbosa, João P Ramos, Raquel Duarte

**Affiliations:** 1. Serviço de Pneumologia, Unidade Local de Saúde de Gaia/Espinho - ULSGE - Vila Nova de Gaia, Portugal.; 2. EPIUnit ITR, Instituto de Saúde Pública, Universidade do Porto, Porto, Portugal.; 3. Instituto de Ciências Biomédicas Abel Salazar, Universidade do Porto, Porto, Portugal.; 4. Instituto Nacional de Saúde Doutor Ricardo Jorge - INSA-Porto - Porto, Portugal.

**Keywords:** Patient reported outcome measures, Qualitative research, Quality of life, Tuberculosis

## Abstract

**Objective:**

Personal experiences, perceptions, and views of patients are crucial in understanding the subjective impacts of diseases. The complexity and duration of tuberculosis treatment impose significant physical, emotional, social, and economic burdens, highlighting the need for person-centered, integrated care strategies that address stigma, fatigue, and accessibility to support well-being. Patient-reported outcomes (PROs) are essential for capturing patient perspectives and improving health care strategies. In this study we explored the multifaceted experiences of patients with tuberculosis, seeking to understand their values and priorities during treatment.

**Methods:**

Semistructured interviews with adult tuberculosis patients were conducted at a referral center for tuberculosis diagnosis and management in northern Portugal. After verbatim transcription and anonymization, thematic analysis was performed.

**Results:**

Seventeen interviews were conducted. Most (58.8%) of the study participants were male, and most had pulmonary tuberculosis. Our thematic analysis identified five PROs: treatment experiences; health-related quality of life; functional status; symptoms and symptom burden; and health behaviors. People with tuberculosis acknowledged the impact of multiple factors on their overall health, particularly the psychological and physical burdens of tuberculosis and its treatment. Several areas for improvement and opportunities for enhanced support were identified, particularly in communication, emotional support, and management of treatment burden.

**Conclusions:**

Our findings highlight the need for tailored PRO measures (PROMs) addressing treatment burden, psychosocial distress, and functional limitations in tuberculosis care. Enhancing communication, psychological support, and multidisciplinary approaches in tuberculosis management could improve patient outcomes and overall well-being. Addressing tuberculosis-related stigma and providing targeted interventions may contribute to a more people-centered approach to care.

## INTRODUCTION

Tuberculosis remains a significant global health challenge, affecting approximately 10.8 million individuals worldwide.^1^ Despite concerted efforts to mitigate its impact, tuberculosis continues to impose a substantial physical, emotional, and social burden on those affected.^2^ ^,^ ^3^ In Portugal, although tuberculosis incidence has gradually declined over recent decades, it remains a pressing public health concern, with an incidence rate of 13.7 cases per 100,000 population in 2023,^4^ underscoring the need for effective and person-centered care strategies. 

Although research on the impact of tuberculosis on quality of life is growing, the specific concerns most valued by people with tuberculosis remain underexplored. The prolonged and complex nature of tuberculosis treatment^5^ often leads to significant emotional and physical fatigue, disrupting daily life.^3^ Although video-observed therapy (VOT) has demonstrated higher patient acceptability than directly observed therapy (DOT),^6^ the treatment burden persists, affecting adherence and overall well-being. 

The social implications of tuberculosis are equally significant, with stigma and discrimination driven by the contagious nature of the disease frequently leading to social isolation and mental health challenges.^7^ The economic burden is also considerable, affecting not only individuals but entire communities.^8^ To ensure equitable access to tuberculosis care and support, a person-centered and integrated approach is essential.^1^

Patient-reported outcomes (PROs) are increasingly recognized as valuable in tuberculosis research.^9^ Defined as “any report of the status of a patient’s health condition that comes directly from the patient, without interpretation by a clinician or anyone else,”^10^ PROs provide critical insights into patient experiences. Although PROs are widely studied in chronic diseases such as cancer, diabetes, and HIV,^11^ their application in tuberculosis remains limited. A systematic review of cancer-related PROs identified symptom control, physical function, and emotional well-being as key patient concerns.^12^ Similarly, tuberculosis research has highlighted the importance of PROs in tailoring treatment and improving patient care.^2^ However, existing patient-reported outcome measures (PROMs) are not specifically designed for tuberculosis, limiting their applicability. 

Despite progress in qualitative tuberculosis research,^13^ most studies on PROs originate from low- or middle-income countries,^14^ where findings are often assessed in isolation. Variability in experiences across different phases of treatment remains underexplored, particularly in high-income settings. The present study addressed this gap by examining the experiences of people living with tuberculosis in Portugal, assessing their values and priorities during treatment. By understanding their perspectives, the present study sought to enhance clinical care, develop person-centered strategies, and improve health outcomes for people with tuberculosis. 

## METHODS

This was a qualitative study conducted from May of 2023 to August of 2024 at the Tuberculosis Outpatient Clinic in Vila Nova de Gaia, Portugal. Located in the largest municipality in northern Portugal, the clinic serves as a referral center for confirmed and probable tuberculosis cases in the region. It also manages cases of multidrug-resistant and extensively drug-resistant tuberculosis in the northern and central regions of Portugal. Ethical approval was obtained from the Northern Regional Health Administration in Portugal (Protocol no. CE/2023/77). 

People ≥ 18 years of age with a confirmed diagnosis of any type of tuberculosis and currently undergoing treatment were recruited through convenience sampling. Eligible participants were contacted by telephone and invited to participate in an interview, either on a prescheduled date or during their routine follow-up visits. All participants received an information sheet detailing the objectives of the study and how the research team intended to use the results. Before data collection, a male health care professional at the clinic provided a thorough explanation of the study, ensuring that participants had the opportunity to ask questions and clarify any concerns. Written informed consent was obtained from all participants. Those who were unable to provide consent or who declined to participate were excluded. 

The semistructured interviews followed a script that was developed by the research team and that was informed by prior studies in the field (see Supplementary material). The script allowed flexibility to explore emerging themes and was pilot tested with five participants, with no subsequent changes thereafter. Participants were asked about various aspects of tuberculosis from their perspective, beginning with their diagnosis, symptoms, and knowledge of the disease. The interviews also explored treatment experiences, including psychosocial implications, challenges related to adherence, and the impact of tuberculosis and its treatment on mental well-being and social interactions. Additionally, participants were asked about their interactions with health care providers, particularly regarding communication patterns and their perceived significance in tuberculosis care. 

All interviews were conducted in person at the clinic by the first author (PV), with no other members of the research team present in the room. The first author received training in qualitative research methods from experienced members of the research team (JPR, MV, and PB). Each session lasted approximately 50 minutes. 

The interviews were audio-recorded (with participant informed consent) and transcribed verbatim into Portuguese. To ensure confidentiality, all transcripts underwent anonymization, with names, geographic data, and other identifiers being removed in order to prevent data triangulation and patient identification. 

A combined inductive and deductive thematic analysis^15^ was conducted by two independent researchers (PV and LLF) until meaning saturation was achieved; that is, when no new themes or subthemes (and their underlying relation) emerged.^16^ Initially, each transcript was independently reviewed, and a preliminary set of themes and subthemes was developed for each transcript. These were subsequently cross-checked and validated by the research team. The analysis was aimed at identifying commonly reported PROs, including treatment experience, health-related quality of life (HRQoL), functional status, symptoms and symptom burden, and health behaviors, while also allowing room for themes outside this framework. The study was conducted in accordance with the Consolidated Criteria for Reporting Qualitative Research (see Supplementary material).^17^

## RESULTS

A total of 17 participants were interviewed. Of those, 58.8% were male, with a mean age of 57.8 ± 14.2 years. Most of the study participants had pulmonary tuberculosis (n = 8), although other forms of tuberculosis, such as pleural, cutaneous, intestinal, bone, and lymph node tuberculosis, were also present. Eight participants were professionally active, and all resided in the Vila Nova de Gaia municipality. Findings were categorized into five main PROs: treatment experience; HRQoL; functional status; symptoms and symptom burden; and health behaviors. Table 1 shows a detailed description of the sociodemographic characteristics of the study participants. 

**Table 1:** Sociodemographic characteristics of the study participants.

Patient	Sex	Age, years	Occupation	Type of tuberculosis
1	Male	70	Retired	Pulmonary
2	Female	67	Retired	Lymph node
3	Male	60	Security guard	Pulmonary
4	Male	78	Retired	Pleural
5	Male	57	Salesman	Pulmonary
6	Female	63	Fishmonger	Cutaneous
7	Male	58	Retired	Pulmonary
8	Male	49	Building contractor	Pleural
9	Male	74	Retired	Lymph node
10	Male	58	Private chauffeur	Gastrointestinal
11	Female	26	Student	Pulmonary
12	Male	45	Street food vendor	Pulmonary
13	Female	76	Retired	Pulmonary, lymph node
14	Female	54	Administrative assistant	Lymph node
15	Female	36	Kitchen assistant	Bone
16	Female	66	Retired	Pulmonary
17	Male	45	Forest ranger	Pulmonary

Participants generally expressed satisfaction with the health care that they received, particularly valuing clear communication from health care professionals (Table 2). Many of the respondents appreciated the support and detailed information provided (see quote number [Q] 1 and Q2; Table 2); however, a subset of patients reported feelings of fear or hesitancy when engaging with health care providers (Q3). The recurring need for more targeted and tuberculosis-specific information was a prominent theme. Diagnostic delays, often attributed to limited awareness among health care professionals, negatively impacted treatment experiences (Q4, Q5, and Q6). Additionally, the burden associated with treatment modalities, particularly in the context of DOT/VOT, was reported to affect adherence and quality of life (Q7 and Q8). 

**Table 2:** Themes and subthemes with illustrative quotes.

Theme	Subtheme	Category	Quote number	Quote
Treatment experience	Health care communication	Patient-doctor relationship	1	I think I’m with people who are from the field. I believe in them. I’m going to do what they’re asking and telling me to do. Because ultimately I’m a layman. And what they’re doing I always believe it’s the best for me. (Patient 3)
			2	They’ve explained everything to me very well and I’m always asking questions when I have doubts. (Patient 14)
			3	Neither outside nor here. I don’t talk to anyone. I deal with it as I can/the best way I can. (Patient 9)
		Limited tuberculosis awareness among health care professionals	4	I was sadder when I sought help from the family doctors. [. . .] When people have a history, they [family doctors] go on the computer and see this and that. I went to the family doctor, and I told him that I’d already had a problem eight years ago. What was I told to do? An expectorant, plus this, plus that. An X-ray and an analysis of the expectoration. In fact, I asked for that myself. And then they gave me the tests to do, and I asked if I could come directly to the tuberculosis outpatient clinic. (Patient 3)
			5	After having clues, let’s say, signs. I think they devalued the signs. And I’m sad because that’s two months I’ve lost. And it’s been two months that aggravated my health. (Patient 3)
			6	It’s not a disease that’s . . . Although it’s being talked about more now. Even in hospitals, staff are tested for tuberculosis and everything. But I think it’s still a bit of a taboo because it’s more closely associated with the lungs. (Patient 14)
	Real-world barriers	Logistical constraints	7	Coming here every day from one end of Gaia to the other, right? In terms of transport, I don’t have my own car. That’s why it takes me two, two and a half hours just to get from here to work and from work to here. And that’s the most painful part. (Patient 14)
			8	It’s tiring. I mean, it’s not tiring, but sometimes my phone runs out of space. As I was saying the day before yesterday, I didn’t do it. I took the medication, but I didn’t get around to making the video. (Patient 15)
Health-related quality of life	Psychosocial impact	Emotional distress	9	I thought everyone in my house was already infected. And I said, oh my God. I said, oh my God, I just got sick, and now I’ve spread the disease to everyone. And I felt sad, I cried and everything. [. . .] I isolated myself from people. I thought that, with this illness, I couldn’t even be around other people. I could only watch them passing through. I’d rather die alone. I don’t know where I got it from, where it came from. But I don’t want to pass it on to someone else. Because it’s too much suffering. (Patient 15)
			10	The only reason I don’t go to my sister-in-law’s house is because of my niece. My niece is ill. I’ve stopped going. I don’t want my little girl to die. (Patient 12)
		Social isolation	11	I wouldn’t be able to socialize with the people I live with at home either. My daughter, my son-in-law, my granddaughter, my wife. And I couldn’t live with them. I had to live in isolation. (Patient 9)
			12	Three months without contact with people. I couldn’t even be with my children or grandchildren. (Patient 13)
			13	It’s a boring phase. Avoiding people. Having to wear a mask. I’m also afraid of passing it on to someone else. And then it never ends. (Patient 5)
	Socioeconomic impact	Financial burden	14	When I was told I had tuberculosis, my work contract had ended, I had no money for the next month, and suddenly I had six months of treatment ahead of me. It was the worst part, but we’ll have to manage with patience. (Patient 11)
		Employment challenges	15	They started to get scared. My colleague, the boss of the van, started to get scared. Go away. Go away, you can’t be here. Go away, you can’t be here. You can’t be here. (Patient 12)
			16	For example, my boss immediately made the comment, “Oh, that’s increasing now because people are living in the neighborhood,” and she immediately asked, “Do Brazilians live in your building?” and I don’t know, what if they do? But they don’t live in the neighborhood. Because I don’t exactly live in a social housing block. (Patient 5)
Functional status	Perceived limitations	Restrictions on daily life and autonomy	17	There’s nothing we can do. We’re trapped. And it’s like being in prison. A prisoner has more freedom. (Patient 3)
	Treatment fatigue	Medication overload	18	I didn’t want to, because of my kidney. I started to think, if they told me to avoid antibiotics, avoid anti-inflammatories, antibiotics, and drink lots of water, and I don’t know what else, and I say, it’s going to finish me off. (Patient 16)
			19	It must be, it must be. Then they were saying, “Oh, so many pills, are you going to take everything?” If you must, you must. (Patient 6)
Symptoms and symptom burden	Psycho-emotional burden	Fear and uncertainty	20	The worry is that I don’t know, I’m going to die. I’m already going to die. I had tuberculosis, and tuberculosis is a disease, isn’t it? It’s not very easy to cure, is it? (Patient 1)
			21	I don’t know if I’m getting better or worse, I don’t know. I take the pills, I go home. (Patient 2)
		Coping with emotional and mental struggles	22	I mean, sometimes I feel really down, don’t I? I feel sad to be here with this, and I don’t know how I got it, you know? (Patient 8)
			23	With the treatment I sometimes thought I’d rather die of the disease than be cured. But then it fell into place, fortunately. (Patient 4)
	Adverse events		24	I’d take them now, and after an hour or an hour and a half I’d go and eat. After a while I’d be vomiting, and I’d throw it all up. (Patient 9)
			25	Then I had to stop because it was damaging my liver. (Patient 13)
Health behaviors	Behavioral adjustments	Precautionary	26	Also, my wife, who didn’t understand, you know? I didn’t have any intimacy with her for almost a month, because I was afraid that I might transmit something. And I’d say to her, we’re not going to be intimate for a while. Until I know the answers, we won’t do anything. (Patient 10)
			27	It was a normal thing. At the time I thought I wouldn’t get too close to people. And always put on a mask if I’m talking to someone. If it’s in the street, for example, I try to steer clear of where there are a lot of people. (Patient 11)
	Health-related decision-making	Health information seeking	28	Yes, I started researching the medication, I started researching certain things, and fortunately the one I got isn’t transmissible. (Patient 4)
			29	And then sometimes you don’t know exactly what’s bad for you and what’s not. Or what’s good for it. Regarding the liver, even on the internet, there isn’t anything . . . There are contradictory things; for example, one website says that oranges cleanse the liver. There’s another that says it’s the worst. The public health lady said that oranges and citrus fruits are not good for you. There’s a problem with food. Information about food. (Patient 5)

Many of the study participants described significant strain on interpersonal relationships, primarily due to fears surrounding the transmission of tuberculosis. Concerns about infecting loved ones, especially vulnerable individuals such as children and the elderly, led to feelings of isolation, loneliness, and even social exclusion (Q9 and Q10). These experiences reinforced internalized stigma and, in several cases, resulted in self-imposed isolation (Q11 and Q12). Although a few of the study participants managed to resume some of the routines of their daily life, most remained fearful of transmitting the disease, which in turn heightened emotional distress (Q13). The reduction in social interactions was compounded by financial instability, with some participants reporting job loss or diminished economic independence (Q14 and Q15). Furthermore, experiences of discrimination and stigma significantly contributed to psychological distress, thereby reducing HRQoL (Q16). 

Tuberculosis symptoms and the treatment process had a tangible impact on participant functional status. Many reported difficulties in performing daily tasks, with a notable perception of lost autonomy (Q17). Concerns regarding the potential impact of tuberculosis on existing comorbid conditions were also voiced (Q18 and Q19). Despite the considerable challenges presented by the disease and its treatment, most of the study participants acknowledged the critical importance of treatment adherence in eventually improving their functional status and alleviating symptoms. 

Participants widely acknowledged the severity of tuberculosis and the considerable difficulty associated with its treatment. Uncertainty regarding treatment outcomes, coupled with the fear of medication side effects, further compounded these challenges (Q20 and Q21). The historical association of tuberculosis with high morbidity added to the emotional distress experienced (Q22 and Q23). Many of the study participants expressed concern that adverse side effects could necessitate treatment discontinuation (Q24 and Q25), thereby prolonging the overall recovery process and exacerbating physical and emotional burdens. 

Participants changed their health habits because of the disease, exhibiting knowledge regarding transmission routes and the need for personal protective equipment to prevent disease spread (including the use of masks and social distancing as protective measures) and emphasizing adaptation to the new context of social interactions. Although these measures were deemed necessary, they often disrupted the spontaneity of social interactions (Q26), contributing to feelings of isolation (Q27). A proactive approach to seeking tuberculosis-related information was common among participants (Q28 and Q29), and many believed that greater knowledge could help alleviate some of the distress associated with the disease. Notably, although participants expressed a desire for guidance on managing lifestyle changes, particularly regarding nutrition, there was a prevailing sentiment that support from health care professionals in this area was insufficient. 

## DISCUSSION

Our qualitative analysis revealed key insights into psychosocial and health care experiences of people undergoing tuberculosis treatment, highlighting their understanding of tuberculosis and its social and economic impacts, as well as the psychological burden of tuberculosis treatment. 

A major PRO identified in the present study centered on treatment experiences, highlighting essential factors for people with tuberculosis, with the need for clear communication playing a crucial role. Although many of the study participants valued the information and support provided by health care professionals-which reassured them throughout the treatment process^18^-others expressed hesitancy or fear in communicating openly, leading to gaps in understanding and emotional distress. This underscores the need for person-centered communication strategies that address medical and psychological needs. Tuberculosis awareness also significantly influenced the overall treatment experience. The literature reiterates that although tuberculosis knowledge is adequate in several health care professional groups, continued efforts are needed to keep this knowledge updated.^19^ Our findings suggest that a lack of awareness among health care professionals contributes to diagnostic delay,^20^ emphasizing the importance of continued training and education for improved PROs. 

Tuberculosis had a profound impact on HRQoL, a dimension known to be deeply affected in people with tuberculosis.^21^ Many of the study participants reported anxiety, fear, and emotional distress, which persisted even after starting adequate treatment. The most significant impact stemmed from limitations in social relationships, particularly with close family members. Many of the study participants isolated themselves from loved ones to prevent transmission, particularly to vulnerable individuals such as children and the elderly, with the sense of isolation-both physical (as a result of mandatory isolation) and social (from stigma and reduced social contact)-worsening emotional distress. 

Tuberculosis treatment also imposed a loss of freedom, restricting their ability to work, travel, or engage in daily activities, further affecting HRQoL. Combined with the physical and psychological burden, these factors had a profound impact on the overall well-being of the study participants, highlighting the need for targeted interventions to enhance patient care.^22^

Financial instability was a major concern, given that prolonged treatment led to loss of employment or income. This financial stress added another layer of difficulty to an already challenging situation, with improved social support and the involvement of family and community members in tuberculosis treatment emerging as critical in addressing this issue.^18^

Stigma and discrimination emerged as another significant factor influencing social interactions and HRQoL, with participants experiencing discrimination from peers, coworkers, and even family members due to misconceptions about tuberculosis transmission. These findings are consistent with those of previous studies highlighting the persistent stigma associated with tuberculosis and its detrimental effects on social well-being.^7^

Functional status was widely recognized as an important outcome, with tuberculosis and its treatment significantly affecting physical abilities. Although the functional impact of tuberculosis sequelae is well documented,^23^ the impact of active tuberculosis on daily living remains underrepresented in the literature. Participants highlighted the burden of symptoms on daily activities, contributing to psychological and physical distress. Despite these challenges, most remained adherent to treatment, recognizing its necessity. Providing adequate support and education on the impact of tuberculosis on daily function could enhance PROs. 

The perception of tuberculosis as a difficult-to-treat disease with a demanding treatment regimen emerged as a key determinant of outcomes, with participants expressing frustration over its complexity and duration. Concerns over medication side effects and uncertainty regarding treatment effectiveness contributed to emotional fatigue, further exacerbating mental health challenges. Although DOT and VOT remain essential for tuberculosis management,^24^ ^,^ ^25^ they impose social, financial, and occupational burdens, for which more patient-friendly approaches should be explored.^26^

Although participants recognized the importance of adherence, fear of potential side effects had a profound impact on their well-being. A structured support network focusing on early identification and management of side effects could ease concerns. Multidisciplinary care, involving physicians, nurses, and social workers,^27^ and, as suggested by our data, the need for nutritional support were highlighted as essential for comprehensive tuberculosis management. 

Changes in health behaviors were also noted, with participants demonstrating increased awareness and understanding of tuberculosis transmission and preventive measures, including mask wearing and social distancing. Although these behaviors are necessary for infection control, they exacerbated feelings of isolation and psychological distress. Knowledge gaps regarding tuberculosis persisted,^28^ with some of the study participants attempting to self-educate, emphasizing the need for structured education and accessible information to improve support and understanding during tuberculosis treatment. 

Our study provides valuable insights into the experiences of people with tuberculosis, capturing multiple dimensions of their health outcomes. Given that most of the studies on similar topics have been conducted in low- or middle-income countries, our findings contribute perspectives from different health care settings, allowing broader comparisons. The wide range of themes explored enabled a comprehensive assessment of participant experiences and needs. However, some limitations must be acknowledged. We were unable to conduct member checking to validate participant responses; this could have strengthened the reliability of our findings. Additionally, because we used convenience sampling, selection bias may have excluded individuals with lower engagement in their treatment, limiting the diversity of perspectives. Recall bias and the self-reported nature of the data may also have influenced responses, as participants may have omitted or emphasized certain experiences. 

The findings of the present study have several important implications for health care providers and policymakers. Each of the identified domains had a significant impact on the overall well-being of patients and could serve as key areas for a more personalized treatment approach targeting enhanced patient outcomes and satisfaction. Improving tuberculosis-related information is crucial not only for people with tuberculosis, given that increased knowledge may ease disease burden, but also for health care professionals, given that enhanced training could increase awareness and lead to improved care. Strengthening patient-provider communication and multidisciplinary support-including nutritional support and psychological support to help patients cope with the emotional distress-could further enhance integrated care. Social determinants must also be considered. Financial assistance and employment protection could help alleviate economic strain, while public health campaigns aimed at reducing tuberculosis-related stigma could foster a more supportive environment. Further research on PROs in tuberculosis care, particularly in high-income settings, is needed. Developing tuberculosis-specific PROMs can promote a person-centered approach, ensuring that treatment aligns with the lived experiences and priorities of people with tuberculosis. 
